# Reversible intracellular acidification and depletion of NTPs provide a potential physiological origin for centuries of dormancy in an Antarctic freshwater copepod

**DOI:** 10.1038/s41598-023-40180-y

**Published:** 2023-08-15

**Authors:** Katherine A. Reed, R. Thomas Williamson, Sung Gu Lee, Jun Hyuck Lee, Joseph A. Covi

**Affiliations:** 1 Department of Biology and Marine Biology, The University of North Carolina at Wilmington, 601 S. College Rd., Wilmington, NC 28403 USA; 2 Department of Chemistry and Biochemistry, The University of North Carolina at Wilmington, 601 S. College Rd., Wilmington, NC 28403 USA; 3 Research Unit of Cryogenic Novel Material, Korea Polar Research Institute (KOPRI), Yeonsu-gu, Incheon, 21990 Korea; 4 Department of Polar Sciences, University of Science and Technology, Incheon, 21990 Korea

**Keywords:** Physiology, Limnology

## Abstract

A great diversity of crustacean zooplankton found in inland and coastal waters produce embryos that settle into bottom sediments to form an egg bank. Embryos from these banks can remain dormant for centuries, creating a reservoir of genetic diversity. A large body of literature describes the ecological and evolutionary importance of zooplankton egg banks. However, literature on the physiological traits behind dormancy in crustacean zooplankton are limited. Most data on the physiology of dormancy comes from research on one species of anostracan, the brine shrimp, *Artemia franciscana*. Anoxia-induced dormancy in this species is facilitated by a profound and reversible acidification of the intracellular space. This acidification is accompanied by a reversible depletion of adenosine triphosphate (ATP). The present study demonstrates that acidification of the intracellular space also occurs in concert with a depletion of nucleoside triphosphates (NTPs) in the Antarctic copepod, *Boeckella poppei*. Like *A. franciscana*, the depletion of NTPs and acidification are rapidly reversed during aerobic recovery in *B. poppei*. These data provide the first comparative evidence that extreme dormancy under anoxia in crustacean zooplankton is associated with intracellular acidification and an ability to recover from the depletion of ATP.

## Introduction

Zooplankton on all seven continents of the world produce embryos that remain dormant for years to centuries before completing their lifecycle^1–6^. The majority of zooplankton found in lakes and estuaries produce dormant embryos^1,7,8^, some of which remain viable for up to 700 years^9^. Similar to the strategy employed by microbial spores or plant seeds, dormancy in zooplankton facilitates temporal dispersion by storing genetic diversity in an “egg bank”^1,7,10^. A great body of research on diverse species demonstrates that zooplankton egg banks perform important ecological and evolutionary functions^1,8,11^. By contrast, research on the physiology of dormancy in crustacean zooplankton is limited. The majority of biochemical and physiological research on dormancy in crustacean zooplankton is restricted to a single species of brine shrimp, *Artemia franciscana* (Kellogg, 1906), the literature on which is extensively reviewed^12–16^. There is a great need for biochemical and physiological studies on distantly related species of zooplankton with a similar ability to suppress development and metabolic processes.

Biochemical studies on hydrated embryos of the brine shrimp suggest that a nearly ametabolic state is achieved in two forms of embryonic extreme dormancy: diapause and quiescence^17,18^. Diapause is a state of developmental and/or metabolic arrest that occurs as part of an obligatory developmental program in a broad diversity of organisms, including *A. franciscana*^12,15^. It is possible to identify embryos capable of diapause by their physical characteristics, but it is not possible to determine if an embryo is in the physiological state of diapause by physical characteristics. An encysted zooplankton embryo is defined as being in diapause when it does not develop under environmental conditions that normally promote metabolism and development^12^. Exit from the diapause state is controlled by exogenous environmental cues like light or oxidative conditions, and the end of diapause is defined by the resumption of development and concomitant metabolic activity^12^. Embryos are referred to as “post-diapause” when development and metabolic activity resume. In contrast with diapause, quiescence is a state of metabolic and developmental arrest that occurs in embryos of *A. franciscana* when environmental oxygen is absent (anoxia)^12–16^. In the simplest of terms, diapause starts because of an endogenous genetic program but quiescence starts because of a specific environmental condition, like the loss of oxygen. Exit from quiescence occurs when environmental oxygen returns, and the embryos go through a period of aerobic recovery. Importantly, quiescence only occurs in zooplankton embryos that also enter diapause. Studies on crustacean zooplankton from inland lakes demonstrate that diapause begins shortly after encysted embryos are released by the female. The brine shrimp, *A. franciscana* is an example of this^15^. Thus, diapause was originally assumed to be a state that precedes quiescence.

Embryos of *A. franciscana* use a profound intracellular acidification (> 1 pH unit) to downregulate metabolic activity under anoxia-induced quiescence^19,20^. A nearly ametabolic state is achieved under anoxia, which simultaneously halts development in the brine shrimp embryo^13,18^. A significant portion of the proton-equivalents causing this acidification are generated by the hydrolysis of NTPs like ATP^12,20^. Upon return of aerobic conditions, NTPs are regenerated and intracellular pH is rapidly restored (alkalinized)^12,19,20^. Development subsequently resumes^20,21^. Prior to the present study, it was unknown if the embryos of other zooplankton species employ transitions in intracellular pH to regulate metabolism and development under anoxia.

Detection of intracellular pH in zooplankton embryos is only possible with phosphorus nuclear magnetic resonance (^31^P-NMR). Premature rupture of the embryonic exoskeleton is usually lethal, and causes developmental arrest when it is not immediately lethal^22^. This is almost certainly because the embryonic exoskeleton provides a critical permeability barrier for hydrophilic substances the embryo must retain, including hydrogen ions and inorganic phosphate^20^. Consequently, the direct measurement of intracellular pH with microelectrodes is not possible. Autofluorescence in the exoskeleton also obscures signals from pH sensitive dyes, thus ruling out the most common indirect measure of intracellular pH. Detection of intracellular pH with ^31^P-NMR is accomplished by observing the resonance signature (chemical shift) of inorganic phosphate (P_i_)^19^. A change in intracellular pH leads to a change in the protonation of P_i_ that is detectable in the chemical shift of the phosphorus atom^19^. Unfortunately, large numbers of embryos are required to generate a detectable P_i_ signal with ^31^P-NMR^19^. Synchrony in development of the embryos also improves homogeneity in NMR chemical shifts (peaks). The need for a large number of synchronized embryos is the reason that physiological and biochemical experiments on *Artemia* were not repeated in other species until the present study.

Copepods are a crustacean zooplankton that provide an ideal model for comparison with anostracans like *Artemia*. The Copepoda are distantly related to the Anostraca^23^, but often have a similar capacity for dormancy^1^. *Boeckella poppei* (Marzek, 1901), is an important and broadly distributed freshwater copepod found in South America, the subantarctic, and Antarctica^24^. Recent research on *B. poppei* demonstrates that this species shares physiological, developmental, and structural characteristics with *A. franciscana*^25,26^. In both species, light and oxygen are required to break out of the endogenously programmed form of dormancy (diapause)^25,27^. Embryos from both species also enter a second reversible state of dormancy (quiescence) when exposed to anoxia^25^. In addition to similarities in dormancy cues, embryos of both *B. poppei* and *A. franciscana* enter diapause as gastrula-stage embryos with incomplete cell membranes, and are protected by an embryonic cyst wall with very similar structural features^26,28^. This high degree of similarity, despite a large evolutionary distance, makes comparisons between *B. poppei* and *A. franciscana* particularly valuable.

*Boeckella poppei* is often the only crustacean zooplankton present in Antarctic lakes^29^. This natural monoculture makes it easy to identify embryos to the species level. Embryos of *B. poppei* also accumulate in the bottom sediment of lakes where they can remain dormant for almost two centuries^3^, and separation of the embryos from lake sediment is simple^25^. Thus, unlike zooplankton found in lakes of more northern latitudes with diverse zooplankton communities, *B. poppei* from Antarctic lakes are available in quantities needed for ^31^P-NMR.

In the present study, ^31^P-NMR is used to observe changes in intracellular pH as embryos of *B. poppei* are exposed to cues that break the diapause state. Changes in intracellular pH are then evaluated in response to anoxia and aerobic recovery from anoxia. Changes in the abundance of other phosphagens, like nucleoside triphosphates (NTPs), are also evaluated, because NTPs decrease dramatically when intracellular pH acidifies in *A. franciscana* under anoxia. The data presented in this study are used to test the hypothesis that extreme dormancy under anoxia in encysted embryos of zooplankton is facilitated by the ability to reversibly acidify the intracellular environment and recover from a prolonged depletion of NTPs like adenosine triphosphate (ATP).

## Results

### ^31^P-NMR with live copepod embryos

**Figure 1:**
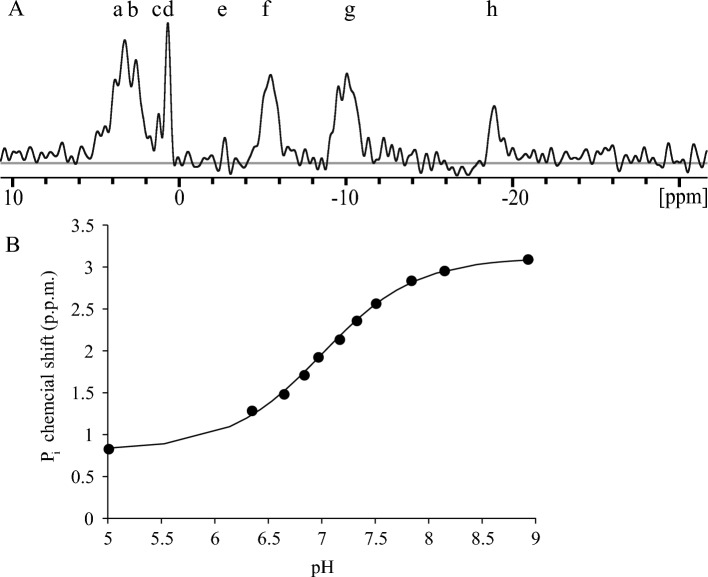
Representative ^31^P-NMR spectrum of *B. poppei* in the ED stage (**A**) and standard curve used to relate the chemical shift of inorganic phosphate to solution pH (**B**) at the ecologically relevant temperature of 4 °C. Predominant chemical shifts for phosphate nuclei are identified by letters: a, phosphomonoesters; b, inorganic phosphate (P_i_); c, Unknown phosphagen 1; d, unknown phosphagen 2; e, unknown phosphagen 3; f, γ-NTP and β-NDP; g, α-NTP and NDP; h, β-NTP. (**B**) The standard curve is described by the equation pH = 6.91 + log[(δ − 0.826)/(3.08 − δ)].

**Figure 2:**
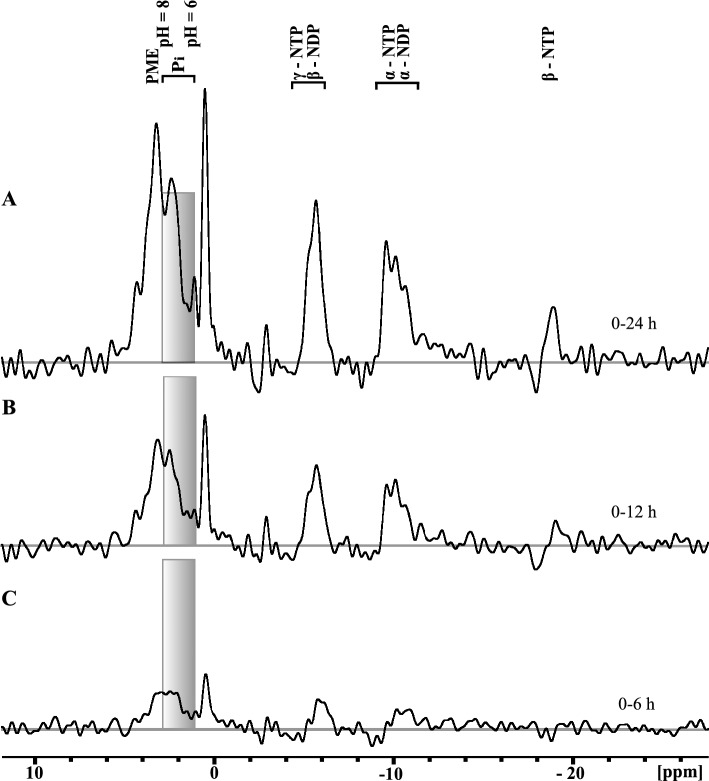
Representative ^31^P-NMR spectra of *B. poppei* in the Early Development stage (ED) summed over (**A**) 24 h, (**B**) 12 h or (**C**) 6 h in artificial freshwater. Predominant chemical shifts for phosphate nuclei are identified above 24 h spectrum. Shaded grey boxes are identical in height to provide a reference for inorganic phosphate (P_i_) peak amplitude and chemical shift. The chemical shift of P_i_ was used to infer intracellular pH.

Phosphomonoesters (PME), inorganic phosphate (P_i_), the three phosphates of NTPs, and the two phosphates of nucleoside diphosphates (NDPs) were detected in Early Development (ED) embryos of *B. poppei* with ^31^P-NMR (Fig. 1A). At least three unidentified phosphagen peaks were also detected (Fig. 1A). Average embryonic intracellular pH was estimated using the chemical shift of inorganic phosphate (P_i_) from 12 h spectra and a standard curve relating the P_i_ chemical shift to solution pH at 277 K (3.85 °C) (Fig. 1B). Values over pH 8 were evaluated qualitatively, because small changes in ppm translate into large pH shifts in this region of the standard curve (Fig. 1B). A comparison of 6 h, 12 h, and 24 h ^31^P-NMR spectra demonstrated that 12 h spectral summations provide the shortest data acquisition time with resolution high enough to identify the chemical shifts of P_i_ and the β-phosphate of NTPs (Fig. 2).

**Figure 3:**
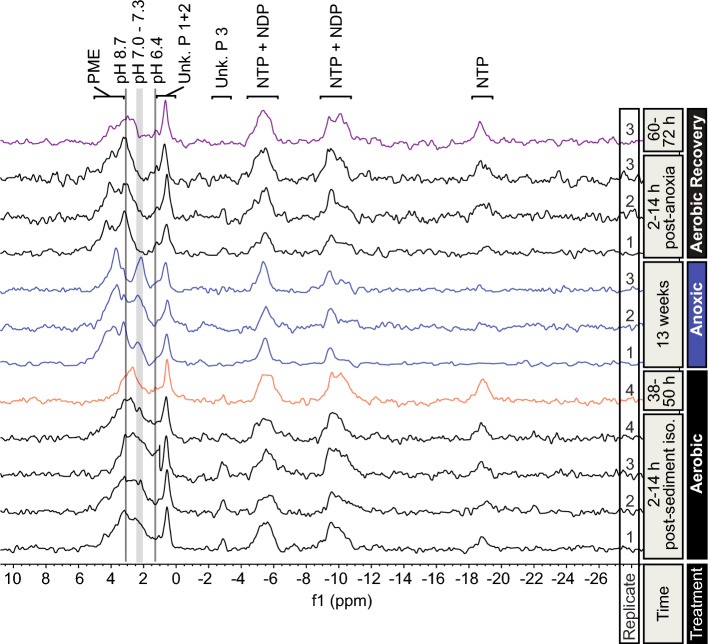
Representative ^31^P-NMR spectra demonstrating changes in pH_i_ and NTPs in live embryos of *B. poppei*. Embryos were isolated from sediment 2 h prior to initiation of the first spectral acquisition. Values for intracellular pH were derived from chemical shift (peaks) for inorganic phosphate, and are indicated with grey bars. Replicate number, time, and treatment type are listed for each spectrum. The first aerobic spectrum acquired for each replicate experiment is placed at the bottom of the graph. Additional spectra acquired for that replicate under anoxia and aerobic recovery are shown above. Three of the four starting aerobic replicates were placed under anoxia for 13-weeks, and subsequently reintroduced to aerobic conditions. Individual representative spectra are provided for time points when n < 3. Spectra for each replicate were summed over 12 h, and therefore represent a time weighted average over 12 h for 1210–1407 embryos. Anoxic exposure commenced at 50 h after isolation from sediment.

The intracellular pH of a population of *B. poppei* embryos was heterogenous during the first 14 h after separation of bulk sediment. Based on the width of the P_i_ peak in ^31^P-NMR spectra, it was inferred that the pH of compartments containing P_i_ ranged from 6.5 to > 8.0 during the 12-h NMR data acquisition (Fig. 3). The chemical shift of the main P_i_ peak during the first 14 h after isolation of embryos from lake sediment indicated a dominant intracellular pH range of 7.4 to > 8.0 (Fig. 3). During the period of 38–50 h of aerobic incubation, a dominant P_i_ peak developed at a chemical shift expected for a solution pH of 7.8 (n = 1) (Fig. 3).

Intracellular pH acidified to 7.17 ± 0.06 (n = 3) after 13 weeks of anoxia, as demonstrated by an upfield (right) shift in the main inorganic phosphate peak (Fig. 3). During the first 14 h of aerobic recovery from anoxia, the main P_i_ peak shifted dramatically downfield (to the left) and merged with the phosphomonoester peak. This shift in the main P_i_ peak indicated that the dominant intracellular pH was > 8 (n = 3) in the majority of embryos during the first 14 h of aerobic recovery from anoxia (Fig. 3). The lower end of the pH range also increased from 6.55 ± 0.08 to 6.83 ± 0.02 (n = 3), as indicated by the downfield (left) shift of valley to the left of the P_i_ peak (Fig. 3). A definitive P_i_ peak was partially resolved from the phosphomonoester peak 60–72 h after the return of aerobic conditions, and this peak indicated a dominant intracellular pH of 7.95 ± 0.32 (n = 2) (Fig. 3). Statistical analysis of pH differences between treatments was not conducted for two reasons: (1) the P_i_ and PME chemical shifts are not resolved under aerobic conditions and (2) the accuracy of pH determination by ^31^P-NMR decreases substantially when greater than pH 8.0.

**Figure 4:**
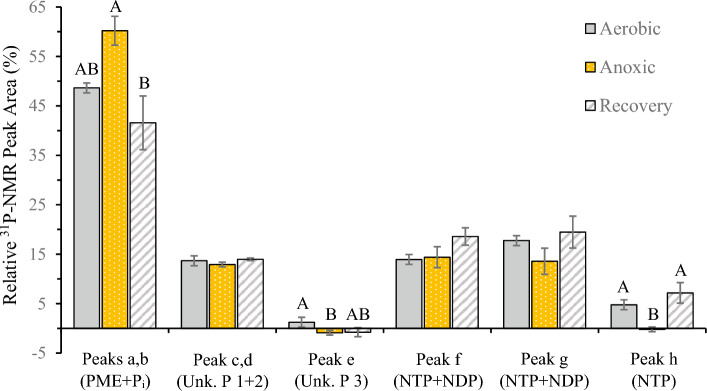
Integration of peaks in ^31^P-NMR spectra of encysted *B. poppei* demonstrate changes in NTPs and phosphomonoesters in response to oxygen availability. Area under peak curves is expressed as a percent of the total area under all curves combined for individual spectra. Negative values are an artifact of baseline noise when peaks are extremely small or absent. Shared letters indicate no significant difference among treatment means for one peak. The absence of letters above bars indicates no significant difference among means. Letter designations for individual peaks match Fig. 1 and acronyms for phosphate containing compounds match Fig. 3.

^31^P-NMR spectra demonstrated that NTP concentration is dependent on oxygen availability (Fig. 3). A broad peak corresponding to γ-NTP and β-NDP was identified between − 4.5 and − 6.6 ppm. This γ-NTP and β-NDP peak narrowed under anoxia and broadened during aerobic recovery from anoxia (Fig. 3), but analysis by integration detected no significant change in total area under the curve for this peak (Fig. 4). A cluster of peaks corresponding to α-NTP and α-NDP was present between − 9.0 and − 11.2 ppm in aerobic spectra. The α-NTP and α-NDP peaks decreased in amplitude under anoxic conditions, but with no discernible change in spectral range (width) (Fig. 3). These qualitative changes to the α-NTP and α-NDP peaks reversed during aerobic recovery (Fig. 3), but analysis by integration detected no statistically significant change in total area under the curve (Fig. 4). A β-NTP peak was identified between − 18.1 and − 19.7 ppm during aerobic incubations (Fig. 3). This β-NTP peak decreased significantly under anoxia (*p* = 0.0370; n = 3) and increased in during aerobic recovery (*p* = 0.0087; n = 3) (Figs. 3 and 4). The β-NTP peak area was the same during aerobic incubation before and after exposure to anoxia (*p* = 0.3799; n = 3) (Fig. 4).

Multiple peaks corresponding to phosphomonoesters were present between 3.1 and 5.0 ppm in ^31^P-NMR spectra of *B. poppei* embryos (Figs. 1 and 3). A single large PME peak developed at 3.5–4.0 ppm under prolonged anoxia (Fig. 3), but the area under the PME peak did not increase significantly under anoxic conditions (*p* = 0.0517; n = 3) (Fig. 4). The area under the PME peaks did, however, decrease significantly during aerobic recovery (*p* = 0.0131; n = 3) (Fig. 4), and the dominant chemical shift for PMEs moved downfield (left) by 0.4 ppm (Fig. 3).

Three unidentified phosphagens were present in aerobic spectra acquired within 50 h of separating embryos from stored lake sediment (Fig. 1). A single small peak produced by unidentified phosphagen #1 was present in all spectra between 0.8 and 1.2 ppm (Figs. 1–3). This peak is in the chemical shift range for P_i_, but did not change under any condition tested. If this phosphagen were inorganic phosphate, it would indicate a stable compartment with a pH of approximately 6.0. Two additional unidentified phosphagens were detected and well resolved in all collected spectra (Figs. 1 and 3). Unidentified phosphagen #2 produced a large peak between 0 and 0.8 ppm (Figs. 1 and 3) that did not change under anoxia (Figs. 3 and 4). Unidentified phosphagen #3 was observed as a strong peak between − 2.5 and − 3.5 ppm (Figs. 1 and 3) in aerobic spectra acquired between 2 and 14 h after isolation of embryos from lake sediment (Fig. 3). This third unidentified phosphagen decreased significantly under anoxia (*p* = 0.0168) (Figs. 3 and 4). It was present in some spectra during aerobic recovery, but no significant increase in peak volume was detected during aerobic recovery (*p* = 0.1647) (Figs. 3 and 4). Unidentified phosphagen #3 was not present in spectra acquired at 60–72 h of aerobic recovery (Fig. 3).

**Figure 5:**
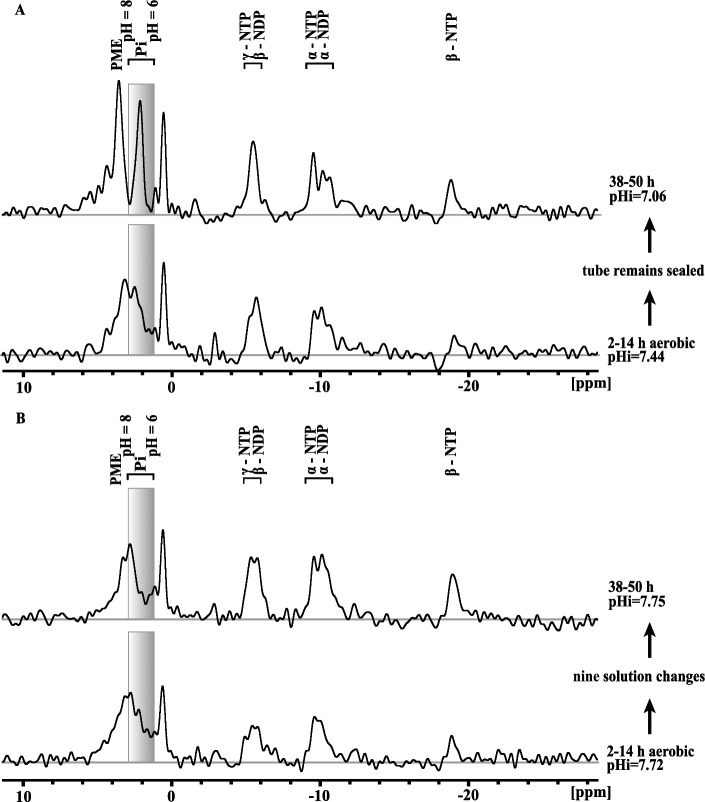
Intracellular pH acidifies and NTPs are depleted in the absence of solution changes when embryos are stored in sealed NMR tubes for 36 h (**A**). Nine solution changes over 24 h between acquisitions of ^31^P-NMR spectra maintained intracellular pH and phosphagen profile normally observed under aerobic conditions (**B**). Embryos placed in NMR tube at T = 0, which is approximately 2 h after isolation of embryos from Antarctic sediments. Shaded grey boxes with identical heights provide reference for inorganic phosphate peak, which is used to infer intracellular pH.

The phosphagen profile in ^31^P-NMR spectra differed between embryos that were left for 48 h in NMR tubes without solution changes (Fig. 5A) and embryos that were in tubes with frequent solution changes (Fig. 5B). Without frequent solution replacement, mean intracellular pH decreased to 7.06 (Fig. 5A). By contrast, intracellular pH within the population of embryos converged on an alkaline pH of 7.75 when solution changes were performed between aerobic spectra (Fig. 5B). The PME peak also increased dramatically and shifted downfield (left) in the absence of solution changes. These changes in PME and P_i_ resonance peaks are similar to those observed under anoxia. However, unlike anoxia, the β-NTP peak is always visible.

### Assessment of embryo viability after ^31^P-NMR

No empty embryonic exoskeletons or nauplii were observed when *B. poppei* were removed from the NMR tubes on the last day of NMR spectra acquisition. Less than 0.002% of embryos were white (dead) when removed from the NMR tubes; 99.998% were orange/red in color. Of the 1210–1407 embryos put into each NMR tube, 83.0 ± 0.8% were in the earliest stage of development (ED), and 16.4 ± 1.1% reached the intermediate stage of development (ID), after 96 d in the NMR tubes. Less than 0.4% reached the prenauplius stage (PN) while in the NMR tube.

After 2 weeks of exposure to light and oxygen in artificial freshwater, 63.5 ± 4.7% of *B. poppei* removed from the NMR tubes remained in the ED stage, and < 0.2% hatched as swimming nauplii. By 28 d of aerobic incubation after removal from the NMR tubes, 37.9 ± 1.4% of ED embryos used in hatching viability tests remained in the ED stage. Another 11.7 ± 1.7% had progressed to the ID stage, 27.7 ± 1.2% were in the PN stage, and 11 ± 2.6% successfully hatched. Development and hatching were still in progress when observation was terminated. Over the 28 d hatching study, 10.1 ± 3.6% of embryos turned white (died). Less than 0.2% of embryos died because of premature emergence during the 28 d hatching test.

## Discussion

Acidification of intracellular pH facilitates extreme metabolic suppression under anoxia in encysted embryos of the brine shrimp, *A. franciscana*^12,13^. For over 40 years, studies on the role of intracellular acidification in other zooplankton species were prevented by a lack of biomass needed for in vivo detection of pH with ^31^P-NMR. This study is the first to overcome the biomass limitation and demonstrate that intracellular pH transitions also occur in embryos of a copepod. Embryos of the Antarctic freshwater copepod, *B. poppei*, can remain dormant for almost two centuries^3^. Part of this resilience is due to their ability to enter a dormant state under anoxia^25^. Much like previous research on *Artemia*, the present study demonstrates that intracellular pH acidifies under anoxia and alkalinizes during aerobic recovery in *B. poppei*. Nucleoside triphosphate concentrations also decrease under anoxia and increase during aerobic recovery in both species. These novel data on a species divergent from *Artemia* support the hypothesis that extreme anoxia tolerance in zooplankton embryos requires the ability to reversibly acidify the intracellular environment and the ability to recover from a prolonged depletion of ATP.

The measurement of intracellular pH in live embryos of zooplankton is challenging. The cells of diapause embryos in zooplankton are partially syncytial, meaning they have incomplete cell membranes^26,28,30,31^. Premature rupture of the embryonic cuticle causes extrusion of cell contents into the environment, which is lethal in both *A. franciscana* and *B. poppei*^22,25^. This sensitivity to rupture prevents the use of microelectrodes. Autofluorescence in the exoskeleton also prevents the use of pH sensitive dyes. Fortunately, in vivo observation of intracellular pH in zooplankton embryos is possible with ^31^P-NMR^19–21^. Using gram quantities of *A. franciscana* provides a strong phosphate signal for ^31^P-NMR with data acquisition times 15 min^19,20^. By contrast, *B. poppei* are only available in mg quantities, and a data acquisition time of 12 h is required to resolve inorganic phosphate and NTP peaks (Fig. 2). The 12 h data acquisition time limits temporal resolution of pH shifts, but allows evaluation of intracellular pH and NTP levels during prolonged exposures to anoxia or aerobic conditions. A benefit of using a smaller quantity of embryos for ^31^P-NMR is that perfusion of the embryos with aerobic media is not required during the acquisition of NMR data. Replacing the aerobic media between sequential 12 h NMR acquisitions maintains aerobic conditions when 1210 – 1407 copepod embryos are used in a single experiment (Fig. 5).

Diapause is a developmentally programmed form of dormancy in zooplankton, and environmental cues are required to “break” out of diapause^16^. Consistent with this, weeks of exposure to light and oxygen in artificial freshwater are required to initiate embryonic development in *B. poppei*^25^. After 5 d of aerobic incubation plus 13 weeks of anoxic incubation in NMR tubes, 83% of *B. poppei* remained in the early development stage. Only 0.3% of embryos progressed past the intermediate development stage in the NMR tubes, and no animals progressed past the pre-nauplius stage. These results are consistent with the previous study by Reed et al.^25^, but improved displacement of oxygen for anoxic treatments decreased embryo development in anoxic treatments in the present study. Some development occurs after nitrogen sparging, because residual oxygen is still present^25^. Embryonic development of both *A. franciscana* and *B. poppei* stops when metabolic use removes the residual oxygen, and true anoxia is achieved^25^. Two weeks after removal from the NMR tubes, 64% of *B. poppei* remain arrested in the early development stage, despite the presence of light and oxygen. After 28 d of exposure to light and oxygen, 38% of *B. poppei* from the NMR tubes remain arrested in the early development stage. Again, these results match those of Reed et al.^25^. Diapause is defined as an arrest of development under conditions that normally promote development. Thus, the observed delay of development under permissive environmental conditions indicates that the majority of *B. poppei* are in diapause when isolated from lake sediments and placed in NMR tubes.

The majority of embryos of *B. poppei* are metabolically active shortly after isolation from lake sediments, including those that appear to be in diapause. Intracellular pH is heterogeneous in a population of embryos for the first 14 h after isolation of *B. poppei* from Antarctic sediments (Figs. 2B, 3 and 5). By 38–50 h after isolation from lake sediments, intracellular pH alkalinizes and heterogeneity of intracellular pH in the population decreases (Figs. 2B, 3 and 5B). Nucleoside triphosphate levels also increase as pH alkalinizes in diapausing *B. poppei* (Figs. 2B, 3 and 5B). The shift in intracellular pH combined with an increase in NTP concentration indicates that the majority of *B. poppei* are metabolically active within 50 h of isolation from lake sediment. This signature of metabolic activity is similar to *Artemia* spp. (Table 1); intracellular pH is alkaline and NTPs are present during early diapause in *Artemia monica* and *A. franciscana*^32^.

**Table 1:** Comparative evaluation of intracellular pH and NTP levels in diapause zooplankton embryos.

		*Artemia franciscana*	*Artemia monica*	*Boeckella poppei*
pH in diapause	Aerobic	≥ 7.9^32^	≥ 7.9^32^	7.8*
	Anoxic	Acidifies^32^	Acidifies^32^	Acidifies*
NTPs in diapause	Aerboic	Present^32^	Present^32^	Present^‡^*^†^
	Anoxic	Decrease^32^	Decrease^32^	Decrease*^†^
	Transition**	nd	nd	Increase^‡^*^†^

NTP, nucleoside triphosphates; **, anoxic to aerobic transition (Aerobic Recovery); nd, no data available; superscript numbers correspond to citations; ‡, Fig. 2; *, Fig. 3; †, Fig. 4.

.

The requirement for ATP during diapause in encysted embryos of crustacean zooplankton requires additional investigation. In *A. franciscana*, ATP levels decrease substantially during diapause while the ATP:ADP ratio decreases during prolonged diapause.^16,33^ In ^31^P-NMR spectra, a decrease in ATP:ADP ratio would appear as a decrease in the β-NTP peak relative to the β-NDP and α-NDP peaks. Contrary to this prediction, all NTP and NDP peaks increase during the first 50 h of development in *B. poppei* (Figs. 2, 3 and 5). Thus, the increase in NTPs in *B. poppei* after isolation from lake sediment is inconsistent with descriptions of prolonged diapause in *A. franciscana*, but may be consistent with *A. monica*.

Active metabolism during diapause occurs in diverse species^14,16^. Future studies on *B. poppei* must determine if diapause involves complete developmental suppression without profound metabolic suppression. Importantly, embryos of *B. poppei* isolated from a lake near the study lake used for the present investigation were determined in a previous study to be up to two centuries old^2^. Two centuries of dormancy in a copepod embryo would require an almost complete metabolic arrest, like that observed in *Artemia* spp. The presence of NTPs in diapause embryos of *B. poppei* under aerobic conditions indicates that metabolic suppression is incomplete (Figs. 2 and 3). Because metabolic processes are still active in diapausing *B. poppei* after many months of storage in native sediments, a secondary process must be responsible for arresting metabolism.

In contrast with diapause, quiescence is a form of dormancy induced by environmental variables, the best studied of which is anoxia^12,13^. Under anoxia-induced quiescence, metabolism is reversibly downregulated, in large part, by a profound acidification of the intracellular space^12,13^. In *A. franciscana*, metabolic heat production drops dramatically under anoxia, and ATP levels are not defended^13,18,21^. As ATP levels decrease in this species, adenosine monophosphate, inorganic phosphate, and organic acids increase^13,21^. As observed with ^31^P-NMR, intracellular pH in *A. franciscana* drops by 1 pH unit within the first hour of anoxia^19,20^. A second phase of slower acidification lowers intracellular pH by another 0.5 pH units over the next day^20^. Observations of intracellular pH in *B. poppei* using ^31^P-NMR demonstrate a less severe acidification of 0.5–0.6 pH units after 13 weeks of anoxia (Fig. 3). However, three factors need to be considered in this estimate of a pH shift in *B. poppei*. First, embryos of *B. poppei* appear to be in diapause, but experiments with *A. franciscana* use post-diapause embryos. This discrepancy between the two species is unavoidable, because a rapid method to terminate diapause in *B. poppei* does not exist. Second, heterogeneity in intracellular pH of *B. poppei* is high after separation from lake sediments (Fig. 2B). This is not surprising, because heterogeneity in intracellular pH measurement with ^31^P-NMR is apparent in post-diapause *A. franciscana*^20^. The heterogeneity could come from variation in the pH or inorganic phosphate content of embryonic compartments, intracellular compartments, or variation in the rate of recovery from hypoxia encountered in the sediment. Variability is not due to developmental stage, because 100% of the embryos were in the ED stage. It is, however, possible that some embryos were activated and exiting diapause, because 83% were in the ED stage at the end of the experiment. Third, the alkaline P_i_ peak and phosphomonoester peak merged under aerobic conditions, making it difficult to determine the exact pH when intracellular pH is > 7.9. The same problem prevented the exact measurement of pH in diapause embryos of *A. franciscana* and *A. monica*^32^. Consequently, measurement of intracellular pH prior to anoxia in *B. poppei* is an approximation, and the 0.5–0.6 pH unit acidification under anoxia is probably larger in post-diapause embryos. A rapid method to break diapause in *B. poppei* is needed to test this hypothesis.

Work on divergent species indicates that anoxia-induced quiescence can occur while embryos of crustacean zooplankton are still in diapause. Interestingly, 83.6 ± 2.3% of control embryos remained dormant during the first 4 days of aerobic incubation of encysted *B. poppei* in culture plates without previous incubation in NMR tubes. The remainder of embryos appear to be in diapause, because it takes weeks of exposure to light and oxygen to break the diapause state in this species^25^. In support of this, > 37% of embryos placed in NMR tubes were still in a state of developmental arrest 28 d after removal from the NMR tubes with incubation under bright light. Thus, it is reasonable to assume that the observed intracellular acidification under anoxia occurred in both diapause and post-diapause embryos in the present study. Acidification under anoxia also occurs in diapause *A. franciscana* and *A. monica*, and NTPs decrease in all three species when diapause embryos are exposed to anoxia (Table 1). These biochemical changes are similar in post-diapause embryos of *A. franciscana* under anoxia^20^.

Importantly, anoxia does not break the diapause state in *Artemia* spp.^32^ Nor does it appear to break diapause in the copepod *B. poppei*. This is indicated by the fact that 83% of *B. poppei* were still arrested in the ED stage of development after 13 weeks of anoxia and 48 h of aerobic recovery (Supplemental Table 1). The lack of influence by anoxia over developemntal arrest in diapause embryos indicates that diapause and anoxia-induced quiescence are separate processes. It was argued previously that metabolism is regulated independently from development in diapausing *Artemia*^32,34^. The authors of the present study argue that the metabolic suppression observed in diapause is enhanced by anoxia-induced quiescence. Support for this hypothesis is provided by the intracellular acidification and depletion of celluar NTPs that occurs when embryos that appear to be in diapause are placed under anoxia (Figs. 3 and 4). The same response was also reported in two *Artemia* species^32^. The profound metabolic arrest that is well documented in quiescent *A. franciscana* would increase survivorship during diapause, and confer an advantage to the diapausing embryo. The present study is groundbreaking in that it provides the first data to support this hypothesis in a copepod.

Alkalinization of intracellular pH during aerobic recovery is faster and greater than acidification under anoxia. Intracellular pH in *Artemia* is completely restored within 20 min of exposure to oxygen^20,21^. Similar to *Artemia*, intracellular pH in *B. poppei* increased by 0.7–1.1 pH units during aerobic recovery (Fig. 3). Unfortunately, it is not possible to observe pH shifts over minutes in *B. poppei*, because observation of pH in this species requires a twelve-hour data acquisition time. However, if intracellular pH had recovered slowly during NMR data acquisition, then the P_i_ peak would appear heterogenous (broad). Importantly, the P_i_ peak is not heterogenous during aerobic recovery (Fig. 3). This indicates that the restoration of an alkaline pH in *B. poppei* is relatively rapid and similar in magnitude to *A. franciscana*.

The acidification of intracellular pH under anoxia in *Artemia* is caused by the net hydrolysis of ATP, accumulation of organic acids, and the dissipation of proton gradients^13,21,35^. Levels of NTPs, which would include ATP, decrease under anoxia and are restored during aerobic recovery (Figs. 3 and 4). Future studies should compare the adenylate status of diapause, quiescent, and developing embryos of *B. poppei* to determine which NTPs decrease during anoxia. Studies with inhibitors of the vacuolar proton ATPase (V-ATPase) and protonophores should also be conducted to determine if proton gradients are dissipated under anoxia in *B. poppei*, because V-ATPase activity is required to restore intracellular pH during aerobic recovery in *A. franciscana*^20^.

Multiple unidentified phosphagens are present in embryos of *B. poppei* immediately after isolation from lake sediment (Fig. 1). The peak for unidentified phosphagen #1 did not change under any conditions and is within the range for inorganic phosphate (Figs. 1 and 3). This peak may represent a stable acidic intracellular compartment. It could also represent a subset of unresponsive embryos. The peak for unidentified phosphagen #2 decreases in amplitude under anoxia and increases during aerobic recovery, but peak area for this phosphagen did not change (Figs. 3 and 4). The chemical shift of this peak is similar to phosphoarginine identified in sponges^36^. A third putative phosphagen is visible immediately after isolation of embryos from lake sediment, but disappears thereafter. Further research is required to conclusively identify unidentified phosphagen peaks in *B. poppei*, which could also include phosphodiesters. It is not possible that any of these peaks were caused by phosphagen release into the medium, because zooplankton embryos capable of dormancy are impermeable to hydrophilic substances. The fact that < 0.002% of embryos died over the course of the 91-day experiments, and almost no ruptured cyst shells were found, indicates that phosphagen signals from dead embryos would be impossible to detect under the signal from living embryos. Furthermore, a ^31^P-NMR test of the medium extracted from a 91-day experiment found no extra-embryonic phosphagen signal.

Extreme dormancy under anoxia is common among inland and coastal zooplankton, but there is little comparative physiological research on dormancy in crustacean zooplankton. The data presented here demonstrate that entrance into anoxia-induced dormancy is accompanied by acidification of intracellular pH and a depletion of NTPs in embryos of the copepod, *B. poppei*. The return of oxygen results in re-alkalization of intracellular pH and the regeneration of NTPs in this species. These physiological events in embryos of *B. poppei* mirror events in diapause and post-diapause *A. franciscana*. Therefore, the authors hypothesize that the evolution of extreme dormancy in crustacean zooplankton is linked to at least two key physiological traits: 1. a capacity to reversibly acidify the intracellular space and 2. the ability to recover from a prolonged depletion of ATP.

## Methods

### Chemicals

All solutions were prepared using ultrapure deionized water (resistivity ≥ 18 MΩ cm at 25ºC). Food-grade table sugar was used to prepare sucrose solutions for density-dependent isolation of dormant zooplankton from sediments. Sterile artificial freshwater (AFW) with a salinity of 0.35‰ was prepared according to the method of Reed et al.^25^ using Instant Ocean® artificial sea salts (Spectrum Brands, Blacksburg, VA, USA). All other chemicals used in the isolation, preincubation and culturing of zooplankton were of ACS grade or higher.

### Measurement of chemical and physical variables at water–sediment interface

A ProDSS YSI multi probe (YSI, Yellow Springs, OH, USA) was deployed to measure water temperature (°C) and salinity (psu) using a Conductivity/Temperature Sensor (626902, ProDSS, YSI, Yellow Springs, OH, USA), pH using a pH Sensor (626904, ProDSS, YSI, Yellow Springs, OH, USA), and dissolved oxygen (mg/L) using a ODO Optical Dissolved Oxygen Sensor (626900, ProDSS, YSI, Yellow Springs, OH, USA).

### Collection, storage, and preparation of zooplankton

Sediment samples containing embryos of the Antarctic copepod, *B. poppei*, were collected in February 2019 by sediment grab sampling from one maritime lake on Barton Peninsula, King George Island, Antarctica, (62.23987° S, 58.74473° W) using a hand-operated stainless steel grab sampler (Product 623–3110, Wildco®, Yulee, FL, USA). See Supplemental Fig. 1 for a description of the workflow. Lake depth at collection sites varied from 2 to 2.5 m, and sediment grab samples were approximately 5 cm in depth and 1 L in volume. At the time of collection, water pH, dissolved oxygen, salinity, and temperature at the sediment water interface were 7.6, 14.7 mg/L, 0.045 psu, and 4.0 °C, respectively. On the day of collection, sediment was mixed to homogeneity and aliquoted in 59 ml Whirl-Pak® plastic bags (Whirl-Pak, Madison, WI, USA). Sediment aliquots were stored at 4 °C and shipped to the University of North Carolina at Wilmington with the assistance of Damco and the National Science Foundation Office of Polar Programs, United States Antarctic Program. The maximum temperature recorded for all samples during shipment was 9 °C. Sediment samples were stored at 4 °C and shielded from light until use. To prevent premature activation of embryo development, work with sediment samples was always conducted at 4 °C under dim red light provided by a 25 W 120 V incandescent bulb with opaque red coating (Feit Electric Company, Pico Rivera, CA). All experiments were conducted between October 2020 and March 2021. Sediment samples used in the present study were the same as those used by Reed et al. 2021 on the same species^26^.

Embryos were isolated from lake sediment on the day of experimentation using the sugar floatation method of Briski et al.^37^ as modified by Reed et al.^25^. All embryos were characterized by color, internal structure, and condition of the cyst wall, as described by Reed et al.^25,26^. Encysted embryos selected for experiments were in the ED stage of development, which is the earliest stage of development where dormancy is observed^25^. Sediment storage for > 8 months under severly hypoxic to anoxic conditions ensured that no subitaneous eggs were used.

### ^31^P-NMR with live copepod embryos

Proton decoupled ^31^P-NMR spectra were acquired at a frequency of 242.97 MHz at a temperature of 277 ± 1 K using a Bruker AVANCE III 600 NMR spectrometer (Bruker, Bellerica, MA, USA). The NMR was locked using 0.05 M triphenyl phosphate in CDCl_3_. A zero ppm chemical shift reference was established using an 85% H_3_PO_4_ standard. Field drift was assessed by the acquisition of 85% H_3_PO_4_ spectra before and after each experiment. Drift was always less than peak resolution of 0.6 Hz. A total of 2048 data points were collected over a spectral width of 82 ppm, and datasets were zero-filled to 32 K. Apodization of free induction decay (FID) was executed with a 35 Hz line-broadening function prior to Fourier transformation. Baseline correction was realized with a 20th order Bernstein polynomial function as integrated in the MNOVA® NMR data processing software package (MestReLabs, Santiago, Spain). NMR spectra were summed in 12 h increments because twelve hours was the shortest data acquisition time that provided optimal resolution of the inorganic orthophosphate (P_i_) and NTP resonance peaks.

Phosphagen identification was accomplished by comparison with published ^31^P-NMR spectra^19–21,38^. Intracellular pH was estimated from the chemical shift (resonance frequency) of P_i_ based on a titration curve produced using a solution of 10 mmol L^−1^ P_i_. The pH of this P_i_ solution was set by the combination of monobasic and dibasic potassium phosphate.

For each ^31^P-NMR replicate, a total of 1210 – 1407 ED embryos were transferred by pipette to a 5 mm diameter glass NMR tube (Sigma-Aldrich Wilmad®, Vineland, NJ, USA). Prior to transferring the embryos, a plug of glass wool (Pyrex® Fiber Glass Roving, 9989 Glass) was placed in the NMR tube and a single piece of glass filter paper was placed on top of the glass wool. The glass filter paper provided a flat surface for the embryos to settle on at the middle of the NMR detection window. After transferring the embryos, additional sterile AFW was added to ensure that there was 0.8 ml of AFW over the glass filter paper. This fluid volume was enough to ensure that the detection window did not contain air. A standard plastic NMR tube cap was placed on the NMR tube and spectral acquisition initiated immediately. The start of the first NMR acquisition was 2–2.5 h after the embryos were isolated from lake sediment. Thus, the start of the first NMR data acquisition was recorded as hour 2 of aerobic incubation.

NMR spectra were acquired under aerobic and anoxic conditions. In brief, embryos were incubated under aerobic conditions in the NMR tube for 48 h (hours 2–50 of aerobic incubation), and then transitioned to anaerobic conditions for a 13-week incubation in the absence of environmental oxygen. Spectra were then collected under anaerobic conditions. Aerobic exposures resumed after the 13-week anaerobic exposure to evaluate recovery from anoxia. Between spectral acquisitions, the clear glass NMR tube containing embryos was stored under the same lighting and temperature regime used for the hatching viability tests (19:5 light:dark; 4 °C).

For the initial aerobic NMR experiments, AFW overlying the embryos was replaced every 2 h between the 2–14 h and 38–50 h data acquisition periods during the day. Tubes were left for 6 h overnight without solution changes. To transition the embryo to anoxic conditions, approximately 0.7 mL of the aerobic AFW was removed from the NMR tube without disturbing the embryos on the glass filter paper. This solution was replaced with the sterile AFW sparged with 99.999% N_2_ to displace oxygen. The NMR tube was then capped with a neoprene gasket secured with Parafilm®, and N_2_ gas was passed into the tube through a 21-gauge needle. A 20-gauge needle was inserted into the neoprene cap for gas outflow. The NMR tube with needles inserted was placed into a glass jar for secondary containment. Both the jar and NMR tube were then purged with 99.999% N_2_ for 5 min to displace oxygen. The headspace of the NMR tube was purged with N_2_ gas for an additional 20 min, and the jar was maintained anoxic by the outflow of N_2_ from the NMR tube. Inflow and outflow gas needles were removed from the NMR tube and Parafilm® was secured completely over the top of the cap. The jar containing the NMR tube was purged with N_2_ for an additional 5 min to displace room air that entered when the needles were removed from the NMR tubes. A metal lid with neoprene seal on the jar was then closed. ^31^P-NMR data collection was conducted after eight weeks of anoxia for a single replicate, after which the jar and NMR tubes were again purged with N_2_ gas using the same method described previously. Spectra collection by ^31^P-NMR was conducted after 13 weeks of anoxia for all replicates.

After the 13-week anoxic test was completed, anoxic AFW was removed from the NMR tube by pipette without disturbing the embryos on the glass filter paper. Sterile AFW equilibrated with room air was then added to the NMR tube. The air-equilibrated AFW was changed five times over 2 h, at which time the first ^31^P-NMR spectra were collected to monitor recovery from anoxia (hours 2–14 of aerobic recovery). NMR spectra were also acquired from 36–48 h and 60–72 h after the return of aerobic conditions. Between spectral acquisition under aerobic conditions, AFW exchanges were conducted every 2 h during the day. The tubes were left for 6 h overnight without solutions changes. Attention was taken to ensure minimal disturbance to *B. poppei* in the tubes during all steps*.*

### Assessment of embryo viability after ^31^P-NMR

After ^31^P-NMR experiments were completed, embryos from the NMR tube were transferred to a petri dish for visual inspection. Developmental stages were assessed based on the criteria of Reed et al. (2018) and empty cyst casings were noted, if present. A subset of embryos was randomly selected for a viability test. In brief, 20–30 ED embryos were placed in each well of a 12-well plastic culture plate containing 1 ml of AFW per well. This provided a total of 287–315 embryos for each hatching test. Plates with embryos were incubated at 4 °C under a 19:5 light:dark regime for 28 days. Development of embryos and hatching of nauplii were recorded weekly.

### Data analysis

A biological replicate for viability tests was considered to be an individual embryo. However, the data were plotted as a percent calculated from observations of all embryos used in each development and hatching test. A random sample of embryos from an individual subsample of stored lake sediment was considered to be a true replicate of the population present in the source lake, and each NMR experiment was conducted using a separate subsample of lake sediment. An individual replicate for NMR is a single assay conducted on a unique sediment subsample.

Peak integration data for ^31^P-NMR were plotted as mean ± standard error of the mean (SEM). One way ANOVA (α = 0.05, JMP 16.0.0 (SAS Institute, Cary, NC, USA) was employed to test the effect of oxygen limitation on the relative abundance of phosphate containing compounds in embryos of *B. poppei.* Brown-Forsythe test was used to test for homogeneity of variance. Tukey–Kramer HSD test was used for multiple comparisons. Intracellular pH values were determined by comparing chemical shift to a generated pH curve and reported as a mean of all replicates.

### Supplementary Information

Supplementary Table 1.

Supplementary Figure 1.

## Data Availability

All data used in calculations and graphs are summarized in Supplementary Table 1.
